# Reworking Nursing Expertise: Directors of Nursing's Tactics to (Re)Connect Knowledge and Power in Hospital Governance

**DOI:** 10.1111/nin.12696

**Published:** 2025-01-08

**Authors:** Dieke Martini, Mirko Noordegraaf, Lisette Schoonhoven, Jet Spits, Pauline Van Bokhorst, Pieterbas Lalleman

**Affiliations:** ^1^ Research Group for Person‐Centeredness in an Ageing Society Fontys University of Applied Sciences Eindhoven The Netherlands; ^2^ Julius Center for Health Sciences and Primary Care University Medical Center Utrecht, Utrecht University Utrecht The Netherlands; ^3^ Utrecht University School of Governance Utrecht The Netherlands; ^4^ Radboud University Medical Centre, Center for Cardiovascular Care Nijmegen the Netherlands

**Keywords:** Board of Directors, Director of Nursing, Governance roles, learning history, nursing expertise, participatory action research, power

## Abstract

Shared governance in hospitals promotes the inclusion of nurses' expertise, knowledge and skills in organisational processes, and nurses increasingly fulfil positions in organisational hierarchies. However, incorporating nursing expertise in strategic governance structures might be complicated, as these structures are primarily linked to managerial and biomedical expertise. Drawing on a Foucauldian perspective on knowledge and power, intertwined and embedded in everyday (inter)actions, we study how newly appointed directors of nursing challenge these dominant ‘modes of knowing’. By focusing on a (Dutch) healthcare organisation, a large academic medical centre, we gained insight into how the history of director of nursing roles relates to how nursing expertise is valued. We gathered qualitative data (from multiple sources) to get close to the daily practices of these directors. In this way, we were able to highlight three *tactics* that enable directors to relate to new ‘knowledge‐power knots’: (1) *positioning*, by creating more unity; (2) *profiling*, by showing significance and (3) *powering*, by being alert and intervening. With these tactics, the directors of nursing try to embed themselves and their expertise in hospital governance. This study contributes to an everyday understanding of power and the tactics that directors of nursing employ as an ongoing practice. This provides practical starting points for embedding nursing in governance and decision‐making.

## 1 Introduction

Shared governance structures in hospitals promote the inclusion of nurses' expertise, knowledge and skills in organisational decision‐making processes (Porter‐O'Grady 2017). Such structures ‘provide pathways for nurses' professional empowerment and influence to shape organisational procedures and policies' (Sundean et al. 2020, 117). Positioning nurses at the strategic apex of healthcare institutions, according to Prybil et al. (2019), positively impacts organisational performance, patient outcomes and population outcomes, as nursing brings a unique and essential set of values and perspectives to address health problems (Thorne 2018). However, although nurses are acknowledged as key players in policy *implementation*, literature shows they are rarely involved in policy development, strategy and decision‐making (Salvage and White 2019; Verhoeven et al. 2023). According to Perry et al. (2023), hospital nurse leaders and nursing staff are often disregarded as active participants in change. Moreover, nursing, as a female‐dominated profession, has been ‘burdened with gender marginalisation and occupational power discrimination within the hierarchies of health care for over a century’ (Sundean et al. 2017, 362). The responsibility, knowledge, skills, accountability and education of nurses, both in terms of status and financial reward, are undervalued in society due to the profession's feminised nature (Clayton‐Hathway et al. 2020). Furthermore, social norms surrounding ‘care work’, and ‘women's work’ position nursing as a less valued form of work (Clayton‐Hathway et al. 2020). As such, nurses' potential to help people ‘live their healthiest lives’ is often underutilized due to organisational barriers or legislation (National Academies of Sciences, Engineering, and Medicine 2021, 363). In addition, nurses are largely unaware of how policy affecting their work is established or that nurses belong at decision‐making tables (Thorne 2022).

Despite these (perceived) disadvantages in the image and position of the nursing profession, the number of nurses in strategic leadership positions in hospitals has slowly increased over the last decades. Nurses take positions as hospital managers and board members, Chief Nursing Officers, Chief Nursing Information Officers and Directors of Nursing. However, incorporating nursing expertise in hospitals' (strategic) apexes is not self‐evident. Hospital governance is dominated by biomedical (Feo and Kitson 2016; Porter‐O'grady 2023) and managerial (Feo and Kitson 2016; Foth, Lange and Smith 2018; Lalleman et al. 2017) expertise. Collaborative and collateral relationships between management and medical staff are firmly rooted in hospitals (Porter‐O'grady 2023; Thorne 2022), forming so‐called ‘knowledge‐power knots’ (Clarke and Newman 2009). Managerial and medical expertise have become the dominant *modes of knowing and acting* (Newman and Clarke 2017).

This study investigates how nurses challenge the dominant modes of knowing when they relate to or become part (or not) of new knowledge‐power knots in the apex of a hospital. We focus on 10 newly appointed Directors of Nursing (DoN) in one large university medical centre in the Netherlands. They are brought into position as part of an organisation‐wide transformation driven by changing societal needs and demands. The hospital strives for a structure that ‘connects, steers based on values, provides space for all relevant perspectives – including nursing – and encourages dialogue and challenges’ (Internal documentation). The DoNs are top‐ranking strategic administrators positioned directly beneath the board of directors and, as such, represent the nursing perspective at the strategic level of the hospital. Responsibilities include but are not limited to setting nursing standards, implementing procedures and policies and developing business plans (Holle et al. 2019). Whilst internationally, DoNs are an established professional group (see e.g., Fineczko et al. 2023; Holle et al. 2019; Lepistö et al. 2017), their appointment is not self‐evident in the Netherlands.

We analyse how the DoNs, as members of interdisciplinary director teams, (attempt to) embed themselves and their expertise in hospital governance and how this relates to the historical role development of DoNs in this hospital. Building on the work of Foucault (2003), we take knowledge (or expertise) and power as closely related and embedded in everyday practices and interactions. This intertwined perspective of power and expertise in everyday practices allows us to understand the tactics of the DoNs better as they attempt to instigate a re‐valuation of nursing expertise. We address the following research question: *Which tactics do newly appointed DoNs employ to (re)work knowledge power balances in a university hospital?* We first elaborate on the literature on power, knowledge‐power knots and tactics.

## 2 Background

### 2.1 Power as Relational and Dynamic

In this study, we approach power as exercised rather than possessed, emerging in relationships that are neither static nor fixed (Foucault 1980). This post‐structural approach to power allows us to focus on shifts in power dynamics in the hospital in two ways. First, power is relational, and power relations are unstable because power is exercised differently, depending on the contexts and the people involved (Foucault 1980). Second, a focus on power and knowledge, as always entangled (Foucault 1980), allows us to investigate and trace the historically constructed nature of specific formations of dominant modes of knowing that have become institutionalised in the hospital but can be contested by other forms of expertise (Newman and Clarke 2017).

### 2.2 Knowledge‐Power Knots

Clarke and Newman (2009) depict the relationship between knowledge and power in so‐called ‘knowledge‐power knots’. The *knot* represents *a* ‘tangled view of multiple threads, rather than a simple, stable and singular relationship between knowledge and power’ (Clarke and Newman 2009, 2). The authors use these knots to unravel and understand how different types of knowledge reform public services. They analyse how the proposition that ‘professionals know best’ is no longer accepted in contemporary societies, leading to a diminishing authority of professionals (Clarke and Newman 2009). We borrow from their work and use the concept of ‘knowledge‐power knots’ to locate multiple forms of knowledge, i.e., (bio)medical, managerial, *and* nursing, and to better understand how they compete to reshape patient care and governance in the hospital. We specifically focus on the *tactics* of the DoNs to accomplish these changes.

### 2.3 Tactics

In the nursing literature, studies refer to tactics as they investigate how nurses work to navigate relations of power (see, e.g., Meng et al. 2015). Furthermore, ample attention is given to the leadership tactics of nurse leaders (see, e.g., Abou Hashish, Alnajjar and Al Saddon 2023; Burkett 2016; Pearson 2020). In organisation sciences, literature on tactics in healthcare organisations focuses on the tactics that (semi)professionals and managers use to achieve or resist organisational change (see, e.g., Ilie and Turel 2020; Kellogg 2018). In this study, in line with Foucault's understanding of power, we employ a post‐structural notion of *tactics* in our analysis of the work of DoNs. Hoff and Kuiper (2021) draw from Foucault's work and define tactics as ‘local, particular instances of power exercise’ (pp. 46–47). Frederiksen, Lomborg and Beedholm (2015) add that tactics are ‘intentional acts that play their part in strategic games in unpredictable ways’ (p. 206).

Focusing on tactics to challenge existing knowledge/power knots in the daily practices of the DoNs as something that they *do* as they rework nursing expertise in hospital governance helps us move beyond a narrative of (historic) powerlessness of nurses toward an understanding of the power that nurses, in various roles, have in their daily work (D'Antonio et al. 2010). We see the re‐organisation of the hospital, including the formation of the interdisciplinary director teams, as creating an organisation that takes multi‐ and interdisciplinary approaches to organising care to meet societal needs and developments such as an ageing population, shortages of nurses, a focus on prevention and rising healthcare costs. In turn, we identify the DoNs tactical work aimed at challenging dominant modes of knowing and obtaining legitimacy as directors. Unavoidably, they must relate to or become part of existing knowledge‐power knots, which are reworked as this happens.

## 3 Methods

We used a ‘learning history’ approach (Roth and Kleiner 1995), a form of participatory action research, to observe and better understand experiences, tensions and successes around introducing the role of DoN in a Dutch hospital. Learning histories are directed at organisational transformation and focus on the ability of organisational members to see that ‘organisational reality is not reified, but rather it is open for reconceptualization through changing old, shared cognitive scripts and learning how to ‘unlearn’ the past’ (Bradbury and Mainemelis 2001, 341). The design incorporates what insiders (practitioners) mostly take for granted, encompassing multiple perspectives from all organisational layers and adding an outsider's (researcher) perspective (Bradbury and Mainemelis 2001). We (D.M., P.L., J.S. and P.B.) documented the experiences and insights of the participants using qualitative research methods and reconstructed them in a ‘jointly told tale’. This created a valuable institutional memory, i.e., a ‘learning history’ (Roth and Kleiner 1995). As reflexive dialogues based on this ‘learning history’ encourage an organisation to move forward (Roth and Kleiner 1995), the design stimulated DoNs and other participants in the hospital to actively engage in consecutive inquiry, learning and reflection around introducing the role of DoN.

### 3.1 Study Setting

This study is part of a national government‐funded research programme called RN2Blend, which investigates differentiated nursing practices in hospital care in the Netherlands (Lalleman et al. 2020). In 2019, there were 69 hospital organisations with 116 locations, including 8 university medical centres and 61 general hospitals in the Netherlands (Steinmann et al. 2022). Dutch hospitals are primarily private, not‐for‐profit organisations typically following a two‐tier governance model. An executive board is responsible for the hospital's daily operations, including financial management, clinical quality and strategy execution. A supervisory board oversees the executive board, ensuring compliance with regulations and the hospital's overall direction. It holds the executive board accountable and is critical in appointing or dismissing its members.

The Dutch healthcare system has a regulated market competition since 2006, which requires care providers and health insurers to negotiate over quality with selected contracting. This created an incentive for hospitals to improve their performance (e.g., Botje, Klazinga and Wagner 2013). Hospitals must comply with national and international quality and safety standards, and the Dutch Healthcare Inspectorate monitors compliance. Patient participation is required by law, in patient councils, patient satisfaction surveys and shared decision‐making structures. Physicians significantly influence hospital governance through medical boards that work closely with the executive board to influence clinical policies, quality standards and staffing decisions (Steinmann et al. 2022). The nursing workforce is typically united in a nursing advisory board or, more recently, in a nursing board; however, nurses are not automatically taken up in hospital governing structures (see, e.g., Verhoeven et al. 2023).

This study took place in a Dutch University Medical Centre (1100 beds), emphasising the work and position of 10 newly appointed DoNs. Data was collected from February to September 2022. During data collection, the hospital prepared for a sizeable organisational transition. Its focus on individual performance and hierarchy did not align with societal desires and developments. Patient care was organised in more than 40 medical departments run by a medical specialist and a business manager. The nursing workforce fell under their responsibility. According to the hospital's governing board this no longer sufficed. The hospital aimed to create an organisation that took a multi‐ and interdisciplinary approach to organising care. In this approach, the expertise of nurses would become equally important as other types of expertise.

After the reorganisation, patient care would be delivered in 10 different patient centres, each governed by an interprofessional board comprising a medical, a business and a nursing director. In addition to the patient care centres, three institutes were to be formed on research, education and patient care. The Institute for Patient Care (IPC) would focus on centralised policies for quality and safety, patient participation, generic nursing processes, care logistics and integral capacity management. The DoN of the IPC would be responsible for all policies of the nursing domain. There was no DoN in the centres for research and education. In the patient care centres, care programmes and pathways would be developed under the dual management of a physician and a clinical nurse specialist. Consequently, nursing expertise would become anchored vertically, in hierarchical structures, and horizontally in care programmes. As such, it would enable significant nursing contributions to patient care processes.

At the time of our research, the reorganisation was still in progress, with both organisational forms existing in parallel. The interprofessional director teams were building their new centres whilst care was still delivered in the old medical departments. During this period of parallel governance structures, the new director teams had no official mandate to make decisions, which temporarily complicated their new role. Furthermore, they still worked their (full‐time) positions in the ‘old’ organisational structure.

### 3.2 Data Collection

Multiple data‐gathering methods were used. All methods helped researchers to get a grip on the work of DoNs. Furthermore, it helped researchers and study participants reflect on their experiences around introducing the role of DoN. The data came from the following sources.

#### 3.2.1 Documents

An archival document analysis of the history of the DoN in the Netherlands was conducted by PvB and consisted of (1) articles from a Dutch nursing periodical *Tijdschrift voor ziekenverpleging* (TvZ) published between 1955 and the present and (2) two hospital policy periodicals, *het Ziekenhuiswezen*, published between 1995 and the present, and *Het Ziekenhuis*, published between 1970 and 1990. In addition, (3) archival documents dating from 1910 until 2000 and (4) 49 annual reports from the hospital dating from 1956 until 2018 were analysed. An archival analysis is useful for capturing professional debates in nursing (McGann 1998); a historical analysis can help draw attention to the manufacturability of current patterns in, in this case, hospital governance (Foth, Lange and Smith 2018). It can make ‘forgotten alternatives’ visible, and, as such, ‘history enables us to realise that what has become our reality was only one option that prevailed by ruling out other options’ (Scott 2007, 28). Furthermore, it gave us insight into the experiences of DoNs over time and helped us draw up a timeline around the hospital's DoNs (Van Bokhorst 2022; Spits et al. 2024).

#### 3.2.2 Shadowing

The first author (D.M.) shadowed eight DoNs for over 80 h. Shadowing is a legitimate method to capture and observe practices that can be difficult to describe in words (Czarniawska 2007). By following the DoNs around ‘like a shadow’, with the opportunity to ask for clarifications, we could view their world ‘from the inside’ (Oldenhof 2017). We came close to their daily work practices and observed how they aimed to embed nursing expertise in the hospital's governing structures. Fieldnotes were written during shadowing and typed out within 24 h after the shadowing had taken place.

#### 3.2.3 Interviews

Two members of the research team (DM, PL) conducted 25 open interviews (45–90 min) from an interpretive perspective aimed at constructing the meaning participants give to their lived experiences (Langley and Meziani 2020). Interview participants were selected through purposeful sampling, either on recommendation from DoNs or based on encounters whilst shadowing. All interview participants were invited to share thoughts, hopes, fears and opinions on the introduction of the DoNs. Interviews were conducted online via MS Teams or in person and transcribed verbatim. Interview participants included nurses, clinical nurse specialists, nurse clinician scientists, physicians, middle managers, executive board members, nursing council members, a nursing science professor and the hospital's Chief Nursing Information Officer.

#### 3.2.4 Focus Groups

Two focus groups (120 min each) were conducted with the DoNs. In both groups, four directors participated. Topics formulated beforehand were (1) collaboration in director teams, (2) finances and (3) the meaning of their role. A fourth topic was added during the focus groups by the DoNs: the visibility of the DoN in the hospital, both in strategic positions and for the nursing profession. This helped us to gain insight into their struggles and successes.

#### 3.2.5 Podcasts

A podcast series of five 40‐min episodes was recorded after a first analysis of the data. Each episode was hosted by a research team member (D.M., P.L.) and handled themes deemed necessary to understand DoN's role better. We selected guests with different backgrounds whom we had spoken to during interviews or encountered whilst shadowing. They were placed together at the podcast table to stimulate rich conversations. The podcast and a written learning history represented a boundary object. A ‘jointly told tale’ distributed in and outside the hospital to stimulate reflection on the role of the DoN and to convey its significance in hospital governance to others. In addition, the podcast was used as a data source in a secondary data analysis session.

### 3.3 Data Analysis

Interpretive description (Thorne 2013) was used throughout the research process. Data analysis was comprised of two stages. The first was in June 2022, when a thematic analysis was conducted in collaboration with selected experts from inside and outside the hospital in a full‐day analysis session. The insiders were a DoN, a medical director and a hospital nursing council member. Outsiders were a professor (emeritus) of management, a professor of public management and a PhD researcher focusing on nursing leadership. Anonymized transcripts of the interviews were divided among the experts beforehand. The session started with a presentation of the (historical) documents analysis by PvB. Then, the experts shared their reflections and associations with the interview transcripts during the data‐analysis session. DM added her reflections based on the field notes whilst shadowing. Others were invited to react based on their expertise. All experts then discussed the findings and identified themes aimed at advancing the position of the DoN. Consequently, DM thematically coded all interview and focus group transcripts and fieldnotes. This led to a written learning history (Martini, Van Bokhorst, et al. 2022) and podcast recordings (Martini, Spits and Lalleman 2022). These ‘learning histories’ were shared in and outside the hospital to instigate reflection and discussion (Bradbury, Roth and Gearty 2015).

Second, in September 2023, the data was revisited by D.M., P.L., L.S. and M.N. to go beyond theming (Thorne 2020), gain rich insights from the data, and engage in the scientific debates on nursing governance. In this stage, the podcast recordings were included as data. We uploaded the recordings in Atlas. ti and coded the audio files. The different data collection methods gained richer insights into the practices related to the positioning of the DoNs (Coté‐Arsenault 2013; Lambert and Loiselle 2008). For instance, interviews and focus groups allowed us to explore why individuals embraced or resisted the role of DoN while shadowing enabled us to observe group dynamics and the way power was distributed. We iteratively analysed data, moving between interviews, focus groups, documents, field notes, and podcasts. Triangulating these different data sources enhanced our understanding of how the DoNs worked to position themselves (Lambert and Loiselle 2008).

We (D.M., P.L., L.S. and M.N.) continuously discussed and compared our findings with the literature, which led us to identify three tactics used by the DoNs to relate to new knowledge/power knots. Differences in opinion sparked discussions on how the ideas generated by the research might impact nurses' daily practices and vice versa. This approach aligns with interpretive description, where the aim is not to codify ‘universal truths’ but to understand how to apply them in unique cases (Thorne 2013).

### 3.4 Ethical Considerations

This study obtained ethical approval (CMO 2019‐5992). Confidentiality was guaranteed to interview‐, and shadow participants, and written approval was obtained. Participants were fully informed about the study before giving consent, and participation was voluntary. Guests featured in the podcast were allowed to review and, if desired, make edits or request the removal of their contributions before the podcast's launch. All data were stored under identification numbers according to scientific rules and legislation.

## 4 Results

During our fieldwork, preparations for the organisational transition were in full swing. Data analysis reveals three ‘tactics’ used by the DoNs to rework nursing expertise in the hospital: (1) *positioning* by creating more unity, (2) *profiling* by showing significance, and (3) *powering* by being alert and intervening. We start with a brief (local) historical perspective on the role of the DoN in this hospital. This will help us better understand the specific (historical) contexts in which the tactics employed by the DoNs to relate to or challenge knowledge/power knots emerged.

### 4.1 Director of Nursing: A (Local) Historical Perspective

Positioning DoNs in Dutch hospital care is not self‐evident, and the instalment of 10 DoNs in one hospital is perceived as a novelty. However, nurses have operated at strategic levels in this hospital before. When founded in 1955, religious nurses were responsible for nursing and management. A ‘Sister‐directress’ (1956–1971), like a director of nursing, supervised general head nurses who, in turn, managed the chief nurses of the various departments under the supervision of a medical director (Van Bokhorst 2022). During a reorganisation in the late sixties, led by medical professionals and ‘business technicians’, nursing knowledge and care were portrayed as less important in shaping hospital governance. Using terms such as ‘technification’, ‘business technicians’ and ‘management’, with their associated male connotations, created an image of men as suitable leaders. Consequently, the female nurses were effectively excluded from the hospital's governance and in 1971, the position of deputy directress was eliminated (Martini, Van Bokhorst, et al. 2022; Van Bokhorst 2022).

Between 1973 and 1991, the nursing staff was supervised by a Head of Nursing Services. However, there was a power imbalance among professional groups (Van Den Bergh‐Braam 1984). In her dissertation, Van Den Bergh‐Braam (1984), nurse, sociologist and Netherlands' first professor of nursing science, explains the position of head nurse in Dutch hospitals. She states that ‘management fails or cannot provide sufficient room for development to the largest group of workers in the hospital, the nurses. The dominance of certain groups is not addressed’ (p. 26). From 1991 until mid‐2000, all care was divided into 12 services and 40 clinical departments were integrated into 10 clusters. The chief medical consultant and the head nurse were jointly responsible for the nursing ward, the workplace and the outpatient clinic (Koesen 2001). Around 2006/2007, however, the hospital faced crises on various fronts: significant financial problems and an extensive health and safety crisis (Wansink and Van den Broek 2011). A new Chair of the Board addressed these issues by ‘putting professionals in the lead’. Those professionals were considered ‘physicians, researchers, and educators’ (Wansink and Van den Broek 2011, 159). This led to a reorganisation in which the nursing professional group again lost its strategic position and became fragmented across 35 departments, with a physician responsible for nursing work.

Retracing the history of nursing governance in the hospital during consecutive reorganisations proved that nurses' positions in hospital governance were vulnerable. This historical perspective helped us better understand and identify the challenges and emerging tactics of the DoNs to rework nursing expertise in the latest hospital reorganisation.

### 4.2 Positioning: Creating More Unity

*Positioning* was the first tactic used by the DoNs. We define positioning as creating spaces for nursing expertise in all layers of the hospital organisation, aiming to create more ‘unity’. This was done in two ways. They aimed to position (a) *nursing as a profession* and (b) *themselves as directors*.

In the ‘old’ organisational structure, nurses were scattered across over 40 medical departments, leading to a proliferation of nursing (leadership) roles in the hospital. One DoN, formerly appointed as healthcare manager on a tactical level, shared the importance of a ‘singular vision’ and ‘joint leadership’ to improve nurses' positions. All healthcare managers in the medical departments in the old organisational structure teamed up to improve the position of nurses.

The moment we started to cross borders between departments and work together with one clear vision and joint leadership, that helped us become a strong group […]. By constantly speaking as one, we found our strength. […] As such, we created opportunities for the nurses in our hospital. We had to unite to get things done because we were not yet in strategic positions. (DoN, reflecting on her former work as a healthcare manager)

The DoN also reflects on the struggles to speak as one:

This was difficult because every medical department had its own needs and interests. However, as a group, we confronted each other and ensured that conflicts of interest were solved. (DoN, reflecting on her former work as a healthcare manager)

The tactic of positioning used by the tactical nurse managers to create a more ‘unified’ professional group was deemed very important by this DoN in instigating change and ensuring strategic roles for nurses:

As healthcare managers, we have been very strong as a group. We have initiated this whole movement with support from the board and finances. And all this without having an official position or anything of the sort. And that is one of the reasons why we are now sitting here as ten nursing directors. We have shown that we can do this. It is unique to have so many directors in one hospital. We made that possible ourselves. This would not have happened without that preliminary phase. (DoN)

In their new roles as DoNs, we observed that they remained focused on working together and creating more unity. For example, together they kept track of and attended all meetings regarding the reorganisation process. The 10 DoNs weekly discussed the attended meetings and their standpoints on current affairs. As such, they were able to respond quickly when the position of the nursing profession was ‘at risk’. For example, they could intervene quickly when profiles for the care programme leaders were presented as medical roles. One DoN stated, ‘We always have to be alert and stay on top of things’. They expressed a ‘disadvantage’ to their medical and financial counterparts whose networks at the organisation's strategic level were much stronger. The DoNs expressed the need for a ‘fair process’.

Others [non‐nursing professionals] quickly arrange something together instead of working through a fair process. I strongly react to that, thinking, how can that be? We agreed on these plans together, didn't we? (DoN)

During our fieldwork, all DoNs fulfilled their position in the ‘old organisation’ (health care manager, nursing director of quality and safety, director of education for healthcare professions, etc.) and their position as DoNs. They ran their departments in the old organisation for over a year, established a centre, and ‘monitored’ the reorganisation to ensure that nurses and nursing knowledge would be positioned accordingly. Because of their busy schedules, they were always ‘switched on’, leaving little time to reflect.

You're putting out a fire on that department at the operational level, then you're dealing with tactical issues, and oh yes, you also must add something at the strategic level. I notice that I have, or take, too little space to prepare for all those roles properly. […] I need to create space to think about what is needed, effectively establish the nursing director's role, and establish person‐centred care within a collegial board. And that's quite complicated. (DoN)

Hence, the DoNs constantly navigated between various organisational layers. They lacked time to reflect on their new roles but simultaneously realised that it was important to create time to synchronise their messages. In one meeting, for example, the DoNs talked about how ‘capacity management’ of the hospital's nursing professionals was discussed by ‘others’. They started to plan how to become involved in these talks of capacity management when one DoN intervened; ‘I share your concerns about how capacity management of nurses is managed in the hospital, but I am not sure if we all agree on how this should be handled. This is a crucial topic that will impact the nursing profession’. Another DoN replies that it might be a good idea first to find consensus on this topic, stating, ‘I should not converse with others based on my viewpoints. We should first find out how we see this as a group’. The DoNs realised the importance of speaking more unitedly, but as indicated, they struggled to find the time to reflect together. As their double roles continued for a year, this struggle did not diminish.

We have our weekly and monthly strategic meetings. But so much is happening in the operation, in the transition operation, that we're mainly focused on that. We're not going one level deeper into where we are now and how we're doing that together. (DoN)

All in all, as 10 DoNs were appointed, their joint reworking of nursing expertise in hospital governance was far from marginal. The DoNs realised the importance of creating more unity. They aimed to get their stories straight and joined forces to position nursing in the reorganisation. However, they struggled to find time to reflect amidst their busy schedules, sometimes felt disadvantaged towards their medical and financial counterparts, and did not always find a more united way to respond or act.

### 4.3 Profiling: Showing Significance

Positioning DoNs acknowledges the value and significance of their input. This input is not secondary but is as important as other input. Positioning the DoNs makes this visible to professionals and patients. (Board member)

Positioning the DoNs thus acknowledged the input of nursing expertise. However, their appointment was not uncontroversial and did not automatically lead to acknowledging the significance of nursing input in an organisation where nursing expertise had been absent at strategic levels for decades. It was unclear how this expertise might contribute to (re)designing healthcare structures. As such, the DoNs used profiling as a second tactic. We define profiling as showing the significance of nursing expertise in (re)designing patient care.

In our first meeting with the DoNs, we proposed to investigate the ‘added value’ of the DoNs as the research project's focus as defining focus together with practitioners is common in learning history research. This led to a discussion; added value implied that the DoNs still had to prove why their position was necessary. This was not the message they wanted to convey. However, their position and the positions of nurses in care programmes were questioned, and nurses' contributions needed to be conveyed. Consensus was reached on the term ‘significance’. If nurses were to take up new positions in the hospital, their significance in the new roles needed to become clear. Carefully choosing the right words showed that the DoNs consciously engaged in their positioning work.

For example, they linked the significance of nursing expertise to person‐centred care.

We take the time to design our centre. We integrate person‐centred care into our plans. This softens the medical/diagnostic approach in which the medical and financial directors reinforce each other. That is why we are positioned, and that is our significance. (DoN)

The DoNs conveyed the significance of nursing expertise in the strategic apex: ‘When I talk with representatives from the current medical departments I constantly explain why it is essential to get dual leadership in patient care programmes’ (DoN). They insisted on nurses' inclusion whenever relevant topics were discussed. e.g., one of the financial directors mentions that ‘when discussing the position of clinical nurse specialists in a department, she (the DoN) clearly expresses her vision’.

Furthermore, the DoNs profiled their ‘implementation power’, emphasising the differences between themselves and physicians. For example, concerning the allocation of certain patient groups:

Two of our centres are closely related. And that is difficult sometimes. Because the medical specialists all have good reasons to want to keep groups of patients in their own centres. But then they keep outbidding each other, and you end up nowhere. That is why it is good that we (DoNs) are present. We represent a different point of view. For example, a certain team cannot be split up and, therefore, needs to be placed in a specific centre. Or just so that we can call out and tell them to stop being difficult. Don't keep talking, do it! (DoN)

The DoNs also worked at strategic levels to convey significance. In the last decades, nursing tasks shifted towards the medical domain. ‘Nurses have lost their craft. We've created a system for nurses that does not necessarily teach them the fundamentals of care’. (DoN) Similarly, the medical domain shaped clinical nurse specialists' work. ‘We gave the job of clinical nurse specialist to the doctors. And they (the doctors) have determined its content’ (DoN). As such, the DoNs aimed to help nursing professionals (re)discover their significance for patient care by conversing with tactical nurse managers, nursing teams, clinical nursing specialists, etc.

It is about going back to the basics. What is nursing all about, and what is your professional identity? Why are you a nurse? That dialogue offers much value. Because sometimes they just don't remember. (DoN)

According to one of the DoNs, it was their job to ‘translate between strategy and practice’. Nurses needed to understand why all the upcoming changes in their work were necessary. By ‘speaking their language and aligning to what is happening in their daily practices’, the DoNs aimed to prepare nursing professionals, recognising the difficulties involved;

We are asking a tremendous amount from nurses in the coming years. And I can be truly shocked by the gap between where we are now and where we want to be. And as directors, we need to go back, back, back to the basics. […] And be satisfied with little steps. This hospital's goals are far too ambitious most of the time. And it is important to adjust them as we go […] and to be proud of small successes. (DoN)

In sum, the DoNs were pivotal in advocating for the significance of nursing expertise within the nursing wards and at tactical and strategic levels. In reaction to the controversy surrounding their appointments, DoNs employed profiling to emphasise the ‘significance’ of their roles and the role of nursing expertise in designing healthcare trajectories. They actively engaged in strategic discussions, advocating for the inclusion of nursing expertise and aimed to connect nursing expertise with policy and practice. Additionally, DoNs worked at operational levels to help nursing professionals rediscover their significance and realign with the evolving healthcare landscape.

### 4.4 Powering: Being Alert and Intervening

Positioning and profiling were important tactics of the DoNs to (re)connect nursing knowledge with power in hospital governance. However, in the transition phase that they were in, talk of change slowly changed into more direct action as people started to see what was going to change for them. Some financial managers and physicians told the DoNs that whilst they [the DoNs] only had things to gain, they would lose things. ‘The current business leaders are losing the nurses and department budgets’. (DoN) In these organisational changes, people questioned decisions and new policies.

The loss becomes evident. Medical department heads and financial managers only see obstacles, not opportunities. […] That is how it has been in this hospital for years. They say; “we have to reconsider this, or we will discuss this again. Send us the documents, and we will think about it”. But we've passed that phase. (DoN)

The DoNs acknowledged that the transition to the centres could only succeed if existing collaborative and hierarchical patterns were ‘broken’. However, they experienced this as a ‘struggle’ in which they used powering as a third tactic to question and/or challenge situations in which their own position, that of nursing knowledge or nurses, was not sufficiently represented. We define ‘powering’ as any direct actions taken by the DoNs to break through existing hierarchical patterns to achieve change. For example, in the new patient care centres, clinical nurse specialists would fall under the responsibility of the DoN who was taken up in an email conversation between different physicians about possibly training a clinical nurse specialist.

To my surprise, I was in the cc but it should have been addressed to me. My job is to connect cure and care, but to these physicians, I am not yet part of the decision‐making table. The DoN took charge of the situation and informed the physicians that the issue was handed over to the clinical nurse specialists. They are specialists in their own profession. And that is the conversation we need to have in this hospital. (DoN)

The DoNs said they were constantly ‘alert’ to situations where they could intervene or adjust. This became apparent when a DoN intervened in a medical department meeting. Physicians discussed a problem in the collaboration between physicians and nurses. Nurses felt forced to admit patients from the emergency room when no beds were available. They indicated this to the medical department, and the physicians discussed how to change it. The DoN intervened;

I said, stop this discussion. I think I should ask the nurses what bothers them. Then I will get back to you with their plans, and you can then agree or not. […] At one point, one physician tells me; “listen to me, I decide whether a patient is admitted to the ward”. And I replied; “that is where you make a mistake. You decide whether a patient has a medical indication to be admitted. That is your expertise. But we decide together if that patient can be admitted to the ward. And if there is no vacancy, you must arrange for your patient to be admitted elsewhere. That's how it should be”.

This DoN reflected on her initial struggles to act accordingly at decision‐making tables, stating that *‘existing hierarchical patterns needed to be broken’*. However, this took some ‘getting used to’;

You have to act as a full member at the table. That works. I have experienced that myself by just taking a seat and speaking up. You can't just sit there and listen. It is hard work. You have to intervene when they talk about your profession. This is my group; I represent them, and you don't anymore. And that is a bit scary at first, but it works. (DoN)

However, they also reflected on their own behaviour and positioning in these changes.

We [nurses] often, perhaps unconsciously, talk about ‘positioning’ and ‘conflict’, which sometimes bothers me. Where does that come from? Doctors don't necessarily see it as a conflict. We might think doctors do not see us as equal, but that doesn't mean it's true. […] However, unconsciously, we tend to operate from a subordinate position, and as such, we make ourselves unequal. (DoN)

This example illustrates an acknowledgement of the hospital's hierarchy. This DoN acknowledged the traditional hierarchical relationships between physicians and nurses as unwanted, yet simultaneously reflected on how nursing professionals reproduced them by operating from a subordinate position. It shows the internalisation of existing hierarchical relationships by nurses. The DoNs used powering to ensure that nursing was taken up in the different layers of the hospital organisation. They expected the need for powering to increase once the reorganisation became official for themselves and other nursing professionals.

They will wake up once we are firmly positioned and can bring our expertise to the table, which will cause friction. […] Once we get dual leadership on the care programs, people must work together in cure and care. On all different levels, we will need leadership from nurses. Moreover, we need physicians to adjust. We have to find a way to work together. (DoN)

In sum, DoNs used powering to ensure nursing influence in the hospital. They were constantly alert and intervened directly when they felt that nursing knowledge or their positions were not adequately represented. The DoNs recognised the need to challenge existing hierarchical patterns, in which physicians and financial directors were used to take charge of situations, and actively sought to ensure their presence at decision‐making tables. However, they also reflected on their behaviour and how they had internalised specific power imbalances. As such, this demonstrated the complexity of navigating power dynamics and fostering collaboration in healthcare settings.

## 5 Discussion

In this study, we used the concept of ‘knowledge‐power knots’ (Newman and Clarke 2017) to better understand how 10 newly appointed DoNs challenged dominant modes of knowing in a university medical centre. We described how they actively gained access to hospital governance and aimed to reshape their positions and profession on several occasions by becoming ‘connectors’ between nursing expertise, hospital governance and practice. We linked their efforts to (re)work nursing expertise in hospital governance to specific local and historical conditions into which their new role was introduced. Our analysis revealed three tactics that the DoNs used to embed nursing expertise in hospital governance: (1) *positioning* by creating unity, (2) *profiling* by showing significance and (3) *powering* by being alert and intervening.

Ideally, shared governance structures in hospitals promote the inclusion of nurses' expertise, knowledge and skills in organisational decision‐making processes (Porter‐O'Grady 2017), and nurses' power within the hospital organisation will increase by adding a nursing perspective on mentorship, structures and processes available to all nurses (Thorne 2018). In practice, appointing DoNs and creating governance structures that promote the inclusion of nursing expertise in the organisation of patient care causes disruption of existing knowledge/power knots in the organisation (Newman and Clarke 2017). This creates spaces for reordering historically grown hierarchical patterns, but new practices do not automatically follow structural changes. Our findings show that specific tactics of DoNs are necessary to change ingrained ‘ways of working’.

Several studies point towards nurses as critical players in policy implementation but as largely absent in the development of policy, strategy and decision‐making (Salvage and White 2019; Verhoeven et al. 2023). The DoNs aimed to create more unity and showed the significance of nursing expertise to position and profile themselves in hospital (governance) structures. They did this by emphasising their implementation power, communication skills and ability to translate between policy, governance and practice. These findings relate to the literature regarding the significance of nurses in healthcare organisations' governance structures as communicators, connectors, advocators and implementers (Foxx and Garner 2021; Sundean et al. 2023). Whilst this significance often remains under the radar (De Kok et al. 2022) or invisible (Allen 2015) in hospital organisations, the presence of the DoNs in hospital governance renders it more visible and, as such, more powerful. Nurses are no longer invited to comment on policy but become part of policy development. As such, nursing expertise becomes represented in hospital policy and the organisation of care. Strategic positions provide nurses with opportunities to influence and improve the delivery of patient care. Furthermore, by linking nursing expertise and person‐centred care, the DoNs worked towards conveying the significance of nursing expertise. The DoNs actively connected nursing expertise and hospital governance.

Nurses are described as being burdened with ‘occupational power discrimination’ within ‘the hierarchies of healthcare’ (Sundean et al. 2017, 362). This is partly in line with our (historical) findings, in which we show how nurses have been largely absent in hospital governance since the introduction of NPM. However, their absence in hospital governance does not make nurses powerless. Nurses actively aimed to reconnect nursing expertise with hospital governance. Nurses' work to create a unified profession, even before they had official positions in governance, contributed to the appointment of then DoNs. This is consistent with research by D'Antonio et al. (2010), which demonstrates that although nurses have historically experienced disadvantages due to invisibility and gender biases, there are many instances in which they show strength, purpose and political action (D'Antonio et al. 2010). Furthermore, it underlines the research of Alvesson and Willmott (2002), López‐Deflory, Perron and Miró‐Bonet (2022) and Schalkwijk et al. (2024), who argue that nurses show agency by actively challenging existing power structures. However, actively challenging power structures in an organisation with ingrained ways of working together was problematic.

DoNs felt they had to stay ‘on top of things’ and were constantly alert about where to intervene. They aimed to do this by creating unity, dividing attention and organising weekly meetings in which important issues concerning nursing were discussed. Martini et al. (2023) refer to this as ‘getting wired in’. They describe how newly appointed nurse clinician‐scientists become part of organising processes by understanding what is going on in the organisation and by relating to others in specific ways to be included. However, in this instance, getting wired in assumed much of the DoNs time. They acknowledged a need to ‘go deeper’ and reflect on their positions and vision together but struggled to find the time to achieve this. As the DoNs were not yet officially appointed, and their positions were contested, they prioritised current issues over long‐term plans and vision. This resonates with the findings of Nicolini and Korica (2021) that CEOs in the British National Health Services work to make room for the things they care about, but that, simultaneously, attention and formal authority coincide. Reworking nursing expertise in hospital governance was an ongoing process that developed over time, and the DoNs felt they could not afford to lose grip. Mainly because they felt that existing hierarchical patterns had been internalised by both nurses and other professionals working in the hospital.

### 5.1 Strengths and Limitations

The combination of multiple qualitative data‐gathering methods gained multilayered insights into the tactics employed by the DoNs that would not have been reached otherwise (Coté‐Arsenault 2013). From this, we were able to derive a broader relevance. However, we consider some spatial and temporal limitations. First, although relations of power and governance are international topics of interest in nursing and DoNs are appointed in many countries, our findings are understood within the Dutch context in which the DoNs were appointed. This potentially limits the generalisation of our findings to other contexts. Second, this study is based on the findings collected at a specific moment in time during the process of reorganisation. However, reworking nursing expertise in hospital governance is a dynamic process that continues to develop over time. As such, further international research into how DoNs challenge dominant modes of knowing in hospital governance is recommended.

### 5.2 Implications and Recommendations

Approaching hospital governance as consisting of knowledge/power knots (cf. Newman and Clarke 2017) and locating tactics to relate to or challenge such knots in the daily practices of DoNs has academic and practical implications. Academically, approaching hospital governance structures as consisting of knowledge/power knots sensitises researchers to existing (historically ingrained) hierarchical structures and patterns and how these might be challenged using specific tactics. As such, it moves away from a dominant paradigm of the history of nursing as relatively powerless (also e.g., D'Antonio et al. 2010, 207). Furthermore, investigating these tactics as something that DoNs do in their daily work, in relation to and with others, provides insights into the ways that power operates and can be challenged within specific socio‐historical contexts (Bradbury‐Jones, Sambrook and Irvine 2008, 264). As such, it can contribute to a better understanding of how nursing expertise might be reworked in hospital governance. Research designs in which researchers can get close to actual daily practices, like the learning history method we have used, can be employed to better understand and improve nurses' positions. In addition, creating a learning history as a joint process with practitioners helps them reflect on their roles and positions in the past, present and future.

More practically, although the results of this study are embedded in specific local historical contexts, some broader (inter)national implications might be derived. Taking up new roles in hospital governance requires nurses to recognise and promote the significance of nursing expertise in hospital governance. This might be done by creating more unity within the nursing profession, actively demonstrating their impact, and learning how to intervene in key decisions. Reworking nursing expertise requires constant alertness from the DoNs and comes with a strong feeling of responsibility to ‘get things right’ for the nursing profession. Their number (10 DoNs) was crucial and might be recommended for other hospital organisations.

Hospitals could work on creating a ‘governance culture’ in which new roles and responsibilities for nurses are formalised both vertically and horizontally in the organisation, with clear opportunities for integrating nurses into strategic decision‐making processes (also see Porter‐O'Grady 2017; Sundean et al. 2020). This acknowledges the value of multidisciplinary inputs, particularly from nursing professionals, to ensure that nursing expertise is not seen as secondary to managerial or biomedical knowledge. Furthermore, board members and managers should actively engage with and challenge nurses to participate in the organisation of nursing care and create spaces for nursing expertise in hospital governance (also e.g., Verhoeven et al. 2022). Finally, being alert to conflicting established interests is essential, as nurses taking up roles in hospital governance will lead to shifts in established power structures. Focusing on enhancing patient care delivery and the significance of biomedical, management and nursing expertise to achieve this will help reshape hospital governance structures.

## 6 Conclusion

This study provides a realistic understanding of how reworking organisational processes and hierarchies plays out. Contrary to the conventional view of nurses as a relatively ‘powerless group’ calling for ‘emancipation’, our findings illustrate how directors of nursing (DoNs) actively sought to reconnect nursing knowledge and power by reworking their positions and profession within hospital governance structures dominated by biomedical and managerial expertise. We show that challenging and/or relating to knowledge/power knots is an ongoing process in which nurses have always played an active role. However, with the appointment of DoNs, this work becomes more visible.

The DoNs positioned themselves as *connectors* between nursing expertise, policy and practice, striving for legitimacy and influence. However, they faced complexities in challenging existing hierarchies ingrained in the hospital organisation and internalised by themselves and other (nursing) professionals. Becoming part of new knowledge/power knots generates a paradox. Shared governance activates differences and divisions between nursing professionals and others. Creating, and relating to new ways of working together is far from easy; it is hard work.

## Ethics Statement

Approval for this study was obtained through the internal review board of the hospital in which the data was collected.

## Conflicts of Interest

The authors declare no conflicts of interest.

## Data Availability

Data available on request due to privacy/ethical restrictions. The data that support the findings of this study are available on request from the corresponding author. The data are not publicly available due to privacy or ethical restrictions.
